# Gamma-Tubulin 1 (TUBG1) Mutation-Associated Lissencephaly and Microcephaly in an Indian Child: A Rare Case

**DOI:** 10.7759/cureus.62749

**Published:** 2024-06-20

**Authors:** Preeti Srivastava, Shikha Swaroop, Kumar Diwakar, Abhishek Jaiswal, Monika Singh

**Affiliations:** 1 Department of Paediatrics, Tata Main Hospital, Jamshedpur, IND; 2 Department of Paediatrics, Manipal Tata Medical College, Manipal Institute of Higher Education (MAHE), Jamshedpur, IND; 3 Department of Radiology, Meherbai Tata Memorial Hospital, Jamshedpur, IND

**Keywords:** early-onset epilepsy, microcephaly, neurodevelopmental disorders, tubg1 mutation, malformation of cortical development

## Abstract

Malformations of cortical development (MCD) are a group of disorders affecting the normal development of the human cortex and are significant causes of delay in psychomotor development and epilepsy in children. Lissencephaly (smooth brain) forms a major group of brain malformations. Microtubules help in the migration of neuronal cells. Defect in tubulin gene alpha-tubulin (TUBA), beta-tubulin (TUBB), and gamma-tubulin (TUBG) leads to defective neuronal migration. This group of disorders is termed as "tubulinopathies." The important genes implicated in causing lissencephaly are LIS1, XLIS, and TUBA1A gene. Recently, a mutation in the TUBG1 gene is associated with it. Here, we report a one-and-a-half-year-old girl with global developmental delay, microcephaly, infantile-onset epilepsy, epileptic spasms, dysmorphism, and motor signs. There was no significant birth history. Neuroimaging (MRI) showed a broad thick gyri and a decreased number of sulci suggestive of lissencephaly/pachygyria spectrum. There was dilatation of the ventricles, and no grey matter heterotopia was noted. Sleep EEG showed multifocal epileptiform discharges. The child was treated with multiple anti-seizure medicines (ASMs). A genetic test, whole exome sequencing, was done to determine the etiology of MCD. A heterozygous missense variation in exon 6 of the TUBG1 gene was identified and reported as a “variant of unknown significance.” Still, because the genotype matched with the clinical phenotype of the patient, it was considered clinically significant. Therefore, a complete diagnosis of TUBG1 mutation-associated cortical malformation (lissencephaly/pachygyria) with microcephaly and early-onset epilepsy was established. TUBG1 mutation is de novo in most cases, but parental testing is recommended. The parents of such patients need to be counseled about the need for prenatal testing and the risk of the disease to siblings. The overall prognosis in such cases is poor because of refractory seizures, physical limitations, and intellectual disability.

## Introduction

Malformations of cortical development (MCD) were first described in 1996 [1]. These are a group of disorders affecting the normal development of the human cortex and leading to a significant delay in psychomotor development and epilepsy in children [2]. They can be divided into two major groups. Group one comprises malformations secondary to abnormal cell proliferation or apoptosis, and group two encompasses malformations secondary to abnormal cell migration [2].

Lissencephaly or “smooth brain” forms a major group of brain malformations that occur because of defective neuronal migration in early pregnancy because of genetic mutations [3]. Apart from genetics, other environmental factors such as hypoxia, infection, radiation, and so on, have a role in the pathogenesis of the disease. Those children affected with the disease have hypertonia, uncontrolled epilepsy, and severe morbidity [4].

Tubulins are a set of proteins present in the cell cytoplasm. Tubulins form the building blocks of microtubules, which are narrow, hollow tubes inside the cell, involved in cell division and cell movement. Microtubules help in the migration of neuronal cells. A defect in tubulin gene alpha-tubulin (TUBA), beta-tubulin (TUBB), and gamma-tubulin (TUBG) leads to defective neuronal migration [5], and this group of disorders is termed as "tubulinopathies." In mammals, gamma-tubulin is highly conserved and is encoded by two genes. Humans have gamma-tubulin 1 and gamma-tubulin 2 (TUBG1 and TUBG2, respectively) genes [6]. TUBG1 is thought to be ubiquitously expressed, whereas TUBG2 is expressed in the brain [7]. TUBG1 encodes TUBG, which is highly expressed in the developing fetal brain as a component of centrosomes [8].

Recent research in the field of genetics shows that novel heterozygous missense variants in the TUBG1 gene cause MCD leading to neurodevelopmental disorders. These disorders manifest as developmental delay, intellectual disability, and intractable epilepsy [9].

Here, we report a case of missense mutation in the TUBG1 gene, which led to lissencephaly presenting with epileptic spasms, spasticity, microcephaly, and global developmental delay.

## Case presentation

**Figure 1:**
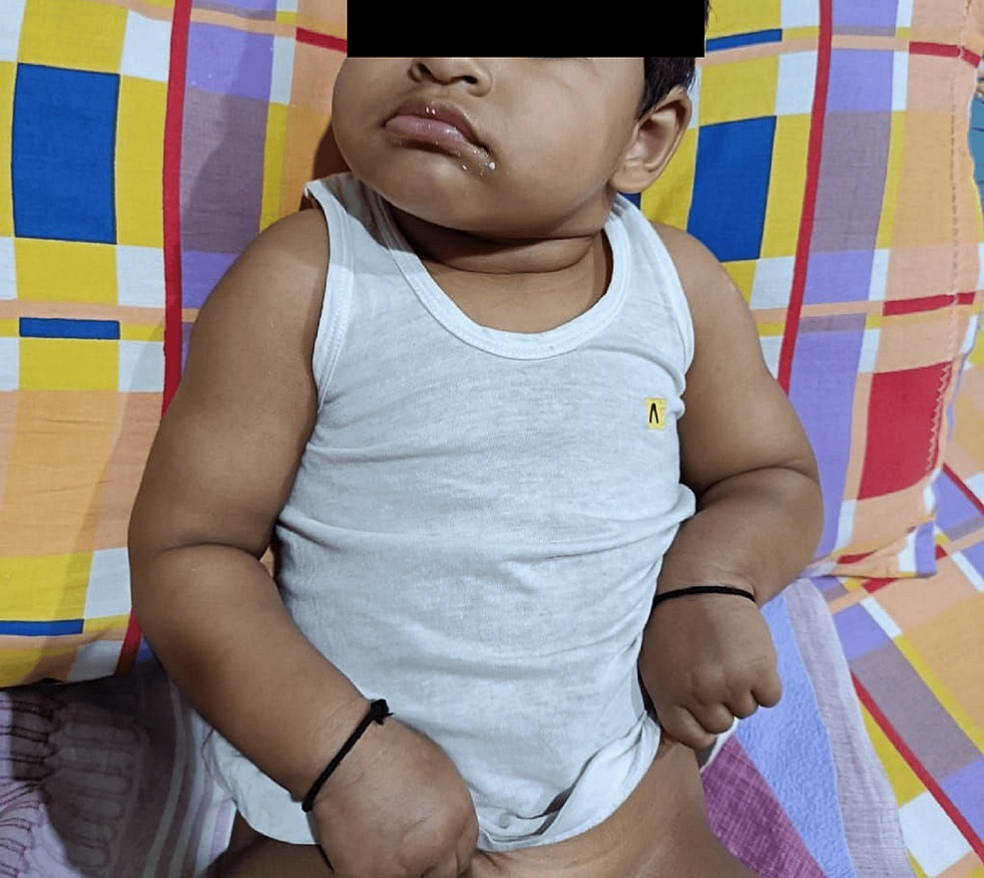
Facial dysmorphism and flexed posture of upper limbs at the elbow and wrists indicating hypertonia.

A one-and-a-half-year-old female, born of nonconsanguineous marriage, presented to our OPD with complaints of global developmental delay and a history of abnormal movements from six months of age with semiology comprising sudden stiffening of limbs with forward bending of the head along with flexion of the trunk. These abnormal events were suggestive of epileptic spasms or myoclonic seizures. Antenatal checkup and delivery occurred outside our hospital. According to the parents, the antenatal checkup and ultrasound were normal, and no abnormalities were reported. Moreover, birth history was insignificant, and there was no admission to the NICU. The index patient was the only child of the couple without any history of sibling loss. There was no history of similar illness in the family. A developmental history revealed that head holding was achieved at five months of age, sitting without support at one year of age and the child could still not crawl or pull to stand or walk independently at one and half years of age. There was no pincer grasp. In language and social domains, the child could make cooing sounds only, did not speak any meaningful words, had inconsistent visual attention, and had an absent social smile. On general examination, dysmorphic features such as anti-mongoloid slant, bulbous nose, and prominent ears were noted. The weight of the child was 9.7 kg (normal for age), and the head circumference was 40 cm (less than the -3z score as per the WHO chart), indicating microcephaly. A neurological examination revealed generalized hypertonia and brisk reflexes with intermittent tonic posturing (Figure 1).

**Figure 2:**
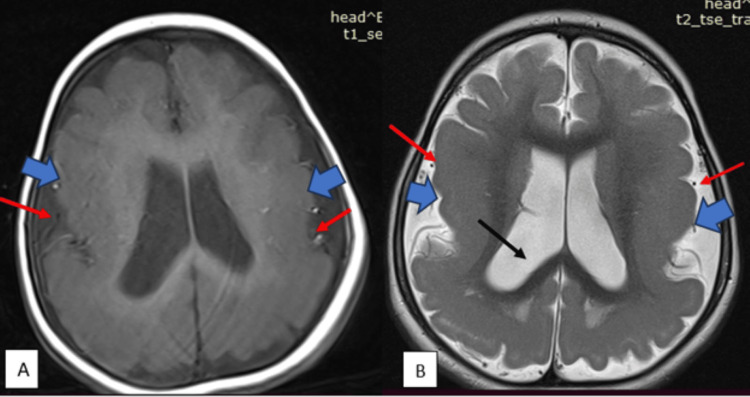
Axial T1W (image A) and T2W (image B) sections of the brain at the level of lateral ventricles show broad thick gyri (blue arrow) and decreased number of sulci (red arrow) suggestive of lissencephaly/pachygyria spectrum. There is dilatation of the ventricles (black arrow). No grey matter heterotopia was noted.

Routine blood tests were within normal limits. The sleep EEG showed multifocal epileptiform discharges predominantly in bilateral fronto-temporo-parietal regions with sleep features such as spindles and K-complexes present bilaterally. An MRI brain showed a broad thick gyri and a decreased number of sulci suggestive of lissencephaly/pachygyria spectrum. There was dilatation of the ventricles, and no grey matter heterotopia was noted (Figure 2). Treatment was started with anti-seizure medicine (ASM) in the form of sodium valproate, followed by clonazepam. There was a reduction in the frequency and intensity of abnormal movements, but they continued to occur daily; hence, oral prednisolone (4-5 mg/kg/day) was started for epileptic spasms. The high-dose oral steroid was gradually tapered and stopped in the next six weeks. The spasms stopped but brief tonic-clonic seizures continued to occur every few days with each seizure lasting less than one to two minutes. Thereafter, topiramate was added to sodium valproate and clonazepam. To establish a complete diagnosis, a genetic test, whole exome sequencing (WES), was done. WES showed a heterozygous missense variation in exon 6 of the TUBG1 gene (chr17: g.42612997A>G; Depth: 81x), which resulted in the amino acid substitution of glycine for glutamic acid at codon 177 (p.Glu177Gly; ENST00000681413.1) and inherited in an autosomal dominant fashion (Table 1).

**Table 1:** Genetic test (whole exome sequencing) report showing a heterozygous missense variation in exon 6 of the TUBG1 gene (chr17:g.42612997A>G; Depth: 81x) that results in the amino acid substitution of glycine for glutamic acid at codon 177 (p.Glu177Gly).

Gene (transcript) #	Location	Variant	Zygosity	Disease (OMIM)	Inheritance	Classification
TUBG1 (+) (ENST00000681413.1)	Exon 6	c.530A>G (p.Glu177Gly)	Heterozygous	Complex cortical dysplasia with other brain malformations 4	Autosomal dominant	Uncertain significance

A review of the literature and reference of the variant to Online Mendelian Inheritance in Man (OMIM) revealed that complex cortical dysplasia with other brain malformations-4 (OMIM#615412) is caused by heterozygous mutations in the TUBG1 gene (OMIM*191135) and inherited as an autosomal dominant disorder. This disorder is characterized by delayed psychomotor development, dysmorphic corpus callosum, MCD, microcephaly, posterior agyria, posterior pachygyria, seizures, spastic tetraplegia, subcortical band heterotopia, and thick cortex with variable severity. It is characterized by early-onset seizures, mostly in the infantile period.

Sanger sequencing of the variant was advised for both parents to classify the variant better; however, the combined cost of this test in both parents was also reasonably high, and the parents did not want to go ahead with the genetic tests but wanted to use their limited resources on the treatment of the child (medicines and physiotherapy). However, the variant was reported as a “variant of unknown significance” (VUS), and the genotype matched the clinical phenotype of the patient; hence, it was considered clinically significant. Hence, a complete diagnosis of TUBG1 mutation-associated cortical malformation (lissencephaly/pachygyria) with microcephaly and early-onset epilepsy was established. The patient has come for follow-up in our OPD at irregular intervals, but seizures are under control on three anti-seizure medicines. The child is undergoing physiotherapy and occupational therapy.

## Discussion

MCD is a rare disorder associated with various genetic mutations. The common clinical features of MCD include motor impairment, intellectual disability, microcephaly, and early-onset epilepsy [4]. Historically, MCD is diagnosed postnatally by MRI brain or by fetal MRI done in the late trimester. Recently, genetic studies and the detection of various mutations, and their causes have been emphasized. Lissencephaly is one of the anatomical phenotypes based on MRI findings.

There are various genes implicated in lissencephaly. The important genes associated with lissencephaly are LIS1 on the short arm of chromosome 17, XLIS located on the long arm of chromosome X, and TUBA1A gene as a third genetic cause of lissencephaly [10,11]. Recently, a mutation in TUBG1 has been seen associated with it [9]. MCDs caused by genetic mutations affecting the function of microtubules are together called tubulinopathies [12].

Tubulin genes are involved in several pathways of cortical development. Mutation in these genes results in cortical malformation by disrupting normal microtubule interaction. The TUBG1 mutation affects neuronal migration. The proliferation of neurons in the fetal brain is not affected [5]. Few TUBG1 mutations have been reported to date. These cases cannot be diagnosed using chromosomal microarrays or biochemical screening for congenital metabolic abnormalities. WES commonly diagnoses such mutations [13]. Motor and cognitive impairments, present in almost all individuals with tubulinopathy, correlate with the severity of brain malformations. Epilepsy varies significantly among affected individuals and does not correlate with the severity of the cortical malformation, the gene involved, or the causative pathogenic variant [14].

In our case, there was a heterozygous missense variation in exon 6 of the TUBG1 gene (chr17: g.42612997A>G; Depth: 81x) that resulted in the amino acid substitution of glycine for glutamic acid at codon 177 (p.Glu177Gly; ENST00000681413.1), inherited as an autosomal dominant condition. Our patient presented with microcephaly and epileptic spasms, which are among the common phenotypes associated with tubulinopathies. Only a few TUBG1 mutations are reported in the literature. Whereas tubulin gene mutations often follow an autosomal dominant mode of inheritance, TUBG1 mutations are almost entirely de novo. The parents were advised Sanger sequencing for the variant to confirm its de novo status as both parents were completely healthy, and no other family member suffered from a similar disorder. However, parents declined further testing because of financial constraints and wanted to focus on the treatment of the child. Hence, segregation analysis could not be done, but the in silico predictions of the variant are damaged by sorting intolerant from tolerant (SIFT), likelihood ratio test (LRT), and MutationTaster2, which is strong (PP3, computational evidence)). The reference codon is conserved across species. The variant was not previously reported in the healthy population (1000Genome, gnomAD, and internal MAF database), which denotes that this is a novel variant (PM2, absent in controls). Although the variant was reported as VUS, it matched the clinical phenotype and, hence, was considered clinically significant.

Genetic testing is important in complex neurological disorders to establish accurate etiological diagnosis. If a significant variant is identified, it is important to analyze and understand the variant, its pathogenicity, its correlation to clinical phenotype, and the risk of similar disease in future pregnancies. If the variant of interest has an autosomal dominant mode of inheritance and if the proband appears to be the only affected family member, molecular genetic testing is recommended for the parents of the proband to confirm their genetic status and allow reliable recurrence risk counseling. If both parents' genetic tests are negative, germline mosaicism cannot be ruled out. Hence, siblings of such patients are at greater risk of having the disease than the general population. Parents of such patients need to be counseled about the need for prenatal testing and the risk of the disease to siblings [15].

Overall prognosis in TUBG1-related MCD is poor because of refractory seizures, physical limitations because of hypertonia, and intellectual disability. Management is supportive and needs a multimodal approach involving pediatric neurologists, occupational therapists, physiotherapists, and so on.

## Conclusions

The widespread availability of genetic tests these days is helping to establish accurate diagnoses in children with complicated neurological disorders. This case report identifies a mutation for a rare neurodevelopmental disorder along with its phenotypic spectrum and dysmorphic features. Such information can guide diagnosis in similar cases. Most of the TUBG1 variants identified are, almost entirely, a de novo mutation, and, hence, it is difficult to implement early screening. Therefore, prenatal diagnosis during pregnancy is an effective means to block the continuation of the genetic factors implicated in the disease. Currently, only symptomatic treatment is available for this condition.
